# Bedside Echocardiography Diagnosis of Tricuspid Valve Infective Endocarditis in the Emergency Department

**DOI:** 10.7759/cureus.29541

**Published:** 2022-09-24

**Authors:** Melissa Bacci, Kishan Patel, Gabriel Cabrera, Eric J Kalivoda

**Affiliations:** 1 Emergency Medicine, HCA Healthcare/USF Morsani College of Medicine GME Consortium, HCA Florida Brandon Hospital, Brandon, USA

**Keywords:** emergency department, tricuspid valve, infective endocarditis, ultrasonography, echocardiography, focused cardiac ultrasound, point-of-care ultrasound

## Abstract

Infective endocarditis (IE) is a rare life-threatening entity that remains difficult to diagnose in the emergency department (ED). Focused cardiac ultrasound (FOCUS) with transthoracic echocardiography (TTE) is an indispensable bedside tool for the emergency physician (EP) to promptly diagnosis and expedite clinical management of IE. This report describes a case in which EP-performed FOCUS led to the early identification of right-sided tricuspid valve IE complicated with septic pulmonary emboli.

## Introduction

Infective endocarditis (IE) is an uncommon potentially fatal condition that presents as a diagnostic challenge in the emergency department (ED). The incidence of IE ranges from 5 to 10 cases per 100,000 person-years, and the incidence is expected to remain stable due to common underlying risk factors of immunosuppression, indwelling medical devices, and intravenous drug use (IVDU) [1-4]. IE carries significant one-year mortality rates approaching 30% [3-4]. Right-sided IE, which affects the tricuspid valve in 90% of cases associated with IVDU, encompasses only 5-10% of all IE diagnoses [3-4]. Septic pulmonary emboli (SPE) are a lethal complication of right-sided IE which further increases the mortality burden [4,5]. Transthoracic echocardiography (TTE) is an invaluable initial diagnostic tool for the emergency physician (EP) to rapidly identify IE [1-4,6,7]. Bedside focused cardiac ultrasound (FOCUS) with EP-performed TTE has previously been shown to be vital for the timely diagnosis of suspected IE [8-15]. This report illustrates the decisive role of EP-performed FOCUS to facilitate the early ED diagnosis and management of right-sided IE notably complicated with SPE.

## Case presentation

A 28-year-old female with a past medical history of seizure disorder and IVDU presented to the ED for evaluation of progressive dyspnea and productive cough of yellowish sputum over the past several days. She also endorsed subjective fever, chills, and generalized malaise. She denied any recent IVDU. She also denied hemoptysis, chest pain, palpitations, syncope, headache, focal neurological deficits, neck pain or stiffness, back pain, abdominal pain, flank pain, nausea, vomiting, diarrhea, or urinary symptoms.

On initial evaluation, the patient was ill-appearing and with the following vital signs: temperature of 39.4°C, blood pressure of 105/64 mmHg, heart rate of 131 beats per minute, respiratory rate 40 of breaths per minute, and oxygen saturation of 90% on room air. She was in moderate respiratory distress, speaking in short phrases, and with diffuse rales on lung auscultation. No cardiac murmurs were appreciated on auscultation, and the extremity examination was negative for edema or asymmetry. Dermatologic examination was unremarkable. The patient repeatedly declined noninvasive positive pressure ventilation but was agreeable to nasal cannula oxygen supplementation, which improved her oxygen saturations. Electrocardiogram revealed sinus tachycardia.

**Figure 1:**
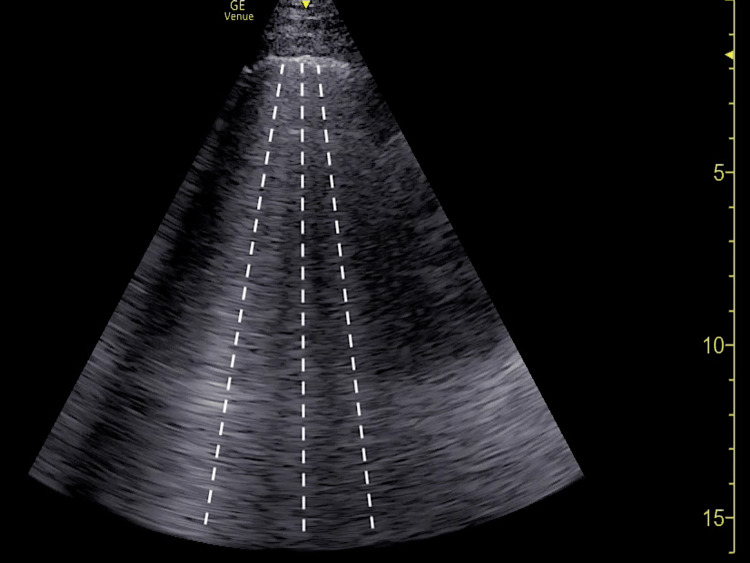
Representative point-of-care lung ultrasound demonstrating diffuse B-line profile (dotted lines) suggestive of multifocal pneumonia versus pulmonary edema.

Bedside lung ultrasound (LUS) and FOCUS were then immediately performed by emergency medicine resident physicians and an ultrasound fellowship-trained attending physician. LUS demonstrated the presence of a diffuse B-line profile suggestive of a multi-lobar pneumonia, although pulmonary edema remained probable (Figure 1). LUS was negative for pleural effusions or any other secondary sonographic findings of pneumonia.

**Figure 2:**
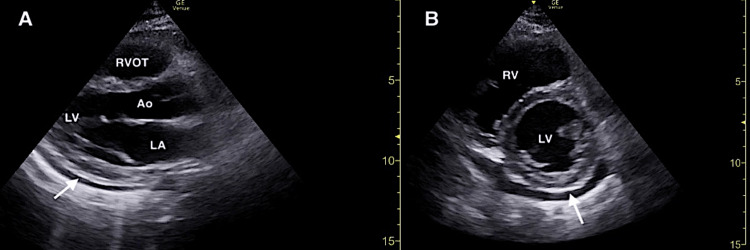
Focused cardiac ultrasound demonstrating a small pericardial effusion (solid white arrow) in the parasternal long (A) and parasternal short (B) windows. RVOT, right ventricular outflow tract; Ao, aortic root; LA, left atrium; LV, left ventricle

**Figure 3:**
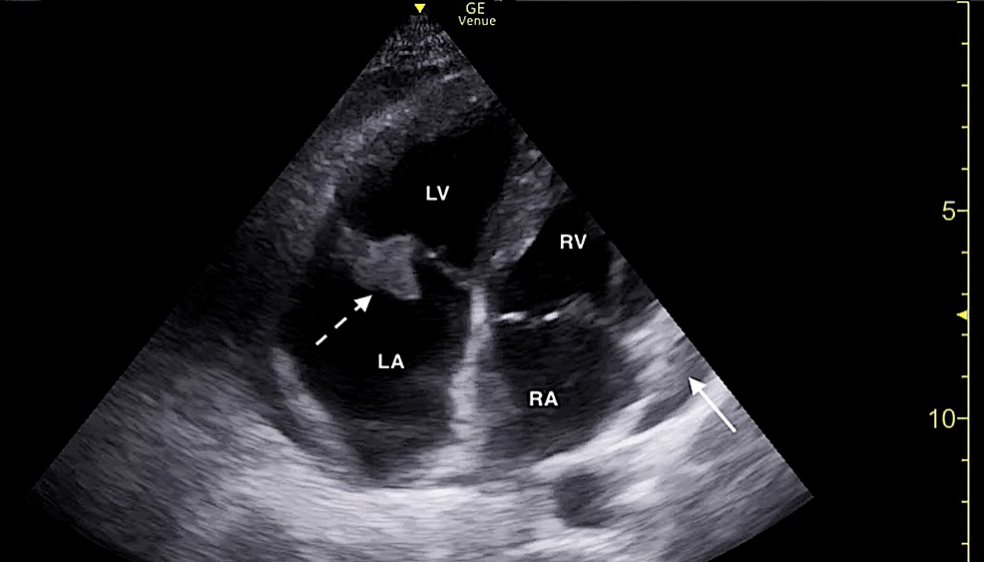
Focused cardiac ultrasound demonstrating a tricuspid valve vegetation (dotted white arrow) and small pericardial effusion (solid white arrow) in the apical four-chamber window. RA, right atrium; RV, right ventricle; LV, left ventricle; LA, left atrium

FOCUS revealed a small pericardial effusion and an irregularly shaped mobile structure located on the tricuspid valve consistent with a vegetation (Figures 2, 3; Video 1). Additionally, FOCUS demonstrated preserved systolic function of the left ventricle, and there were no convincing findings of right ventricular strain.

**Video 1:** Focused cardiac ultrasound demonstrating a tricuspid valve vegetation and small pericardial effusion in the apical four-chamber window.

Laboratory and radiographic studies, blood cultures, intravenous antibiotics, antipyretics, and intravenous fluids were ordered for the empiric treatment of suspected sepsis secondary to right-sided IE likely complicated by a multi-lobar pneumonia.

**Figure 4:**
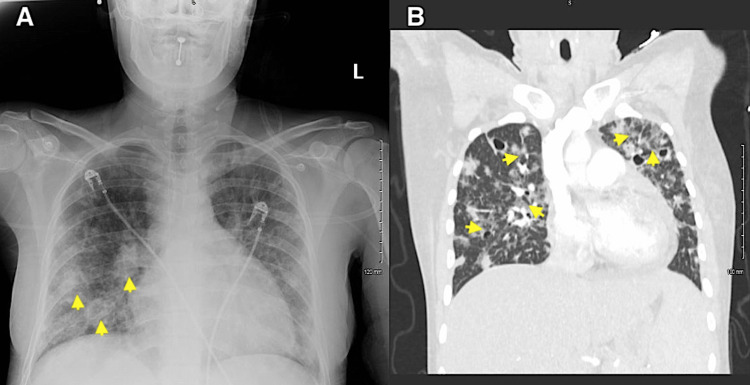
Portable chest radiography demonstrating mild cardiomegaly and infiltrates (yellow arrowheads) representative of septic emboli. (B) Computed tomography pulmonary angiography demonstrating scattered patchy ground-glass infiltrates in a perihilar distribution and numerous nodular densities with central cavitation throughout both lungs (yellow arrowheads), representative of septic emboli.

Portable chest radiography revealed mild cardiomegaly and infiltrates of likely septic emboli (Figure 4A). Computed tomography pulmonary angiography (CTPA) was subsequently obtained, which demonstrated scattered patchy ground-glass infiltrates in a perihilar distribution and numerous nodular densities with central cavitation throughout both lungs, likely representing septic emboli (Figure 4B). CTPA was unremarkable for pulmonary embolism or thoracic aortic dissection/aneurysm.

Laboratory analysis was notable for an initial elevated lactic acid of 4.0 mmol/L (reference range: 0.4 - 2.0 mmol/L), white blood cell count within normal limits at 9.0 x 10^3^/uL (reference range: 4.0-11.0 x 10^3^/uL), and an elevated C-reactive protein at 15.80 mg/dL (reference range: 0-0.30 mg/dL). Cardiac biomarkers were also elevated with troponin I of 0.079 ng/mL reference range: (reference range: 0-0.045 ng/mL) and N-terminal pro B-type natriuretic peptide (NT-proBNP) of 1,066 pg/mL (reference range: 0-125 pg/mL). The initial rapid COVID-19 test was negative.

The patient’s clinical respiratory and hemodynamic status remained stable during the ED course. She was admitted to the intensive care unit (ICU) for further management of tricuspid valve IE complicated with SPE. Formal cardiology-performed TTE and transesophageal echocardiography (TEE) confirmed a small-to-moderate pericardial effusion, a tricuspid valve vegetation measuring 1.2 cm x 1.8 cm, moderate-to-severe tricuspid regurgitation, and moderately increased right ventricle systolic pressure of 54 mmHg. Blood cultures were positive for methicillin-susceptible *Staphylococcus aureus*. The patient had a lengthy hospital course complicated by COVID-19; however, no invasive cardiac interventions were necessitated. The patient was ultimately discharged on a prolonged course of intravenous antibiotics.

## Discussion

The diagnosis of IE in the ED is challenging due to the widely varied constellation of non-specific symptomatology [1-3]. Although fever and malaise are the most common presenting features that should initiate clinical suspicion of IE in higher-risk patient populations, the definitive diagnosis is contingent on satisfying the modified Duke criteria [1-3]. One of the major elements in the Duke criteria comprises a positive echocardiogram for IE, which is defined as either the presence of an intra-cardiac mass or abscess on valves or supporting structures, new or acute worsening of valvular regurgitation, or new disruption of a prosthetic valve [1-3]. Hence, it is imperative that EPs recognize the potential advantages of implementing an initial bedside ultrasound evaluation in cases when there is clinical concern for IE [8-15]. The diagnostic performance of TTE for IE has been reported with a sensitivity of 61% and a specificity of 94% [1-3,6,7]. The TTE sensitivity increases to 84% with IE vegetations larger than 10 mm [6]. EPs can also employ FOCUS to evaluate for crucial IE complications such as cardiogenic shock or acute heart failure secondary to severe valvular regurgitation [3]. Additionally, our case of right-sided IE involved complication with SPE, in which we utilized point-of-care LUS that was consistent with a multi-lobar pneumonia. LUS has previously been proposed as an adjunct ED diagnostic for accurately identifying sonographic findings of pneumonia, including B-line patterns, pleural effusions with or without associated consolidations to indicate empyema, subpleural consolidations with pleural irregularity, and/or air bronchograms [16]. The described case highlights the importance of EPs incorporating point-of-care ultrasound (POCUS) into clinical ED practice for the management of IE and its lethal complications.

Emergency POCUS is a central component of emergency medicine residency training [17,18]. POCUS holds multiple key advantages for EPs in the emergent setting given its accessibility, non-invasive characteristics, and inherent capability for repeated serial bedside examinations. Most EPs obtain a high level of competency in the core content of cardiac and LUS techniques [17-20]. However, it is notable that POCUS of cardiac valvular assessment and pneumonia, both of which are essential to the evaluation of IE, are considered advanced skills; in our opinion, these specific topics should be emphasized more during training to improve the likelihood of future use in clinical practice [17,18]. Advanced EP-performed POCUS certainly has an evolving role within the ED scope of practice for the management of particular cardiothoracic emergencies such as IE.

## Conclusions

IE and its associated complications are often fatal if not recognized and treated promptly. An initial bedside EP-performed TTE can be instrumental in identifying abnormal valvular lesions and/or acute valvular regurgitation concerning for IE. EPs are uniquely positioned to conceivably diagnose IE with POCUS early in its course and subsequently expedite care. Future studies are warranted to investigate the clinical impact and diagnostic accuracy of EP-performed TTE for IE.
